# Chromodomain Helicase DNA–Binding Proteins and Spermatogenesis: Current Advances

**DOI:** 10.1111/andr.70232

**Published:** 2026-04-10

**Authors:** Mingrui Zhang, Di Liu, Guoqiang Liu, Minggang Zhu, Feng Pan

**Affiliations:** ^1^ Department of Urology Union Hospital, Tongji Medical College, Huazhong University of Science and Technology Wuhan China; ^2^ Urology Department of Guangzhou First People's Hospital Guangzhou China; ^3^ Urology Department of Nanjing First Hospital Nanjing China; ^4^ Hubei Jiangxia Laboratory Wuhan China

**Keywords:** chromatin remodeling, chromodomain helicase DNA‐binding (CHD) protein, male infertility, spermatogenesis, therapy

## Abstract

**Background:**

Male infertility is a prevalent clinical condition, with approximately one‐third of cases classified as idiopathic, frequently stemming from impaired spermatogenesis because of dysregulated gene expression. Chromodomain helicase DNA‐binding (CHD) proteins are central chromatin remodelers that orchestrate this epigenetic regulation. For instance, smoking‐induced hypermethylation of the *CHD5* gene promoter is associated with impaired semen parameters.

**Objective:**

This review aims to summarize the roles and mechanisms of CHD proteins in spermatogenesis, explore their relationship with male infertility, and discuss their potential as diagnostic and therapeutic targets.

**Methods:**

A comprehensive literature review was conducted to synthesize current findings on the expression and function of CHD proteins across different stages of spermatogenesis.

**Results:**

Research has shown that CHD proteins are expressed in the testis and play crucial roles in multiple stages of spermatogenesis, from spermatogonial stem cell (SSC) maintenance to spermatid maturation, making them promising targets for understanding and treating male infertility.

**Conclusion:**

CHD proteins represent important epigenetic regulators in spermatogenesis and may serve as valuable biomarkers or therapeutic targets for male infertility.

**Abbreviations**
ADA2: adenosine deaminase 2
Ccnb1: cyclin B1
Ccnd2: cyclin D2
CHD: chromatin helicase DNA‐binding protein
CSTF3: cleavage stimulation factor subunit 3
Dmrt1: doublesex and mab‐3‐related transcription factor 1
GDNF: glial cell line‐derived neurotrophic factor
H3K4me3: trimethylation of lysine 4 on histone H3 protein subunit
HORMA: Hop1p, Rev7p, and MAD2 domain
IHH: male hypogonadotropic hypogonadism
INO80: INOsitol requiring 80
ISWI: imitation switch
NOA: non‐obstructive azoospermia
NuRD: nucleosome remodeling and deacetylase
OCT4: octamer‐binding transcription factor 4
PARP1: poly (ADP‐ribose) polymerase 1
PHD: plant homeodomain
PLZF: promyelocytic leukemia zinc finger protein
Prm1/2: protamine 1/2
SALL4: Sal‐like 4
SANT: Swi3, Ada2, N‐CoR, TFIIIB
SNF: sucrose non‐fermentable
SSC: spermatogonial stem cell
SWI: switch
Tnp1/2: transition protein 1/2

## 1 Introduction

Infertility is a global health issue affecting approximately 8%–12% of couples, with male factors contributing to infertility in approximately half of these cases [1, 2]. In 60%–75% of male infertility cases, the underlying cause remains unidentified [3, 4]. Impaired spermatogenesis is a major contributor to male infertility. Spermatogenesis is a complex process involving cell differentiation, including the proliferation and differentiation of spermatogonia, spermatocyte meiosis, and spermatid metamorphosis [5, 6, 7]. This process requires precise regulation of gene expression, particularly during spermatid maturation, when transcriptional activity gradually ceases and posttranslational modifications become crucial [8, 9, 10].

Chromatin remodeling has been shown to play a key role in spermatogenesis [11, 12]. In eukaryotes, ATP‐dependent chromatin remodelers are generally classified into four major families: the switch/sucrose non‐fermentable (SWI/SNF), imitation SWI (ISWI), chromodomain helicase DNA‐binding (CHD) proteins, and the INOsitol requiring 80 (INO80) family [13, 14, 15, 16, 17]. CHD proteins are distinguished by the presence of two N‐terminal chromodomains that function as interaction surfaces for various chromatin components [18]. They alter chromatin structure by regulating the accessibility of nucleosomal DNA, thereby influencing gene expression [19, 20]. The CHD protein family consists of nine proteins and can be classified into three subfamilies on the basis of domain homology: CHD1–2, CHD3–5, and CHD6–9. They are expressed at different developmental stages and in different tissues and are involved in diverse biological processes [21, 22].

In recent decades, an increasing number of studies have clearly shown that chromatin remodeling defects are closely related to male infertility, among which chromatin remodeling related to CHD proteins accounts for a considerable proportion [23, 24, 25, 26]. On the basis of previous findings, we will systematically explore the roles and functions of the three subfamilies of CHD proteins across different stages of spermatogenesis and elucidate their regulatory mechanisms. In addition, this review elucidates the relationship between CHD proteins and male infertility, thereby providing a conceptual foundation for future therapeutic development.

## 2 Role of CHD Proteins in Spermatogenesis

An increasing number of studies have demonstrated that various members of the CHD protein family play crucial and distinct roles throughout spermatogenesis, ranging from spermatogonial stem cell (SSC) maintenance to spermatid maturation, as summarized in Table 1.

**TABLE 1:** Functions of chromodomain helicase DNA‐binding (CHD) proteins in various stages of spermatogenesis.

Stages of spermatogenesis	Roles of CHD proteins	References
Spermatogonial stem cell stage	CHD2 increases chromatin accessibility and mRNA stability to support SSC self‐renewal. CHD4 regulates the expression of Dmrt1 and PLZF to ensure SSC survival. CHD8 maintains the proliferative activity of spermatogonia	[12, 25, 30, 34, 36, 43]
Spermatocyte stage	CHD7 and CHD8 ensure the smooth progression of spermatocyte meiosis. CHD7 participates in non‐homologous end joining to facilitate DNA double‐strand break repair. CHD8 ensures normal meiotic DSB formation	[43, 85]
Spermatid stage	CHD1 and CHD5 facilitate spermatid metamorphosis and maturation. CHD1 ensures normal acetylation of histone H4 and chromatin condensation. CHD5 ensures the regular shape of the sperm nucleus and highly condensed chromatin	[28, 29, 30, 31, 41, 73, 91, 102, 103]

Abbreviations: Dmrt1, doublesex and mab‐3‐related transcription factor 1; PLZF, promyelocytic leukemia zinc finger protein; SSC, spermatogonial stem cell.

### 2.1 Role of the CHD1 Subfamily (CHD1–2) in Spermatogenesis

**FIGURE 1:**
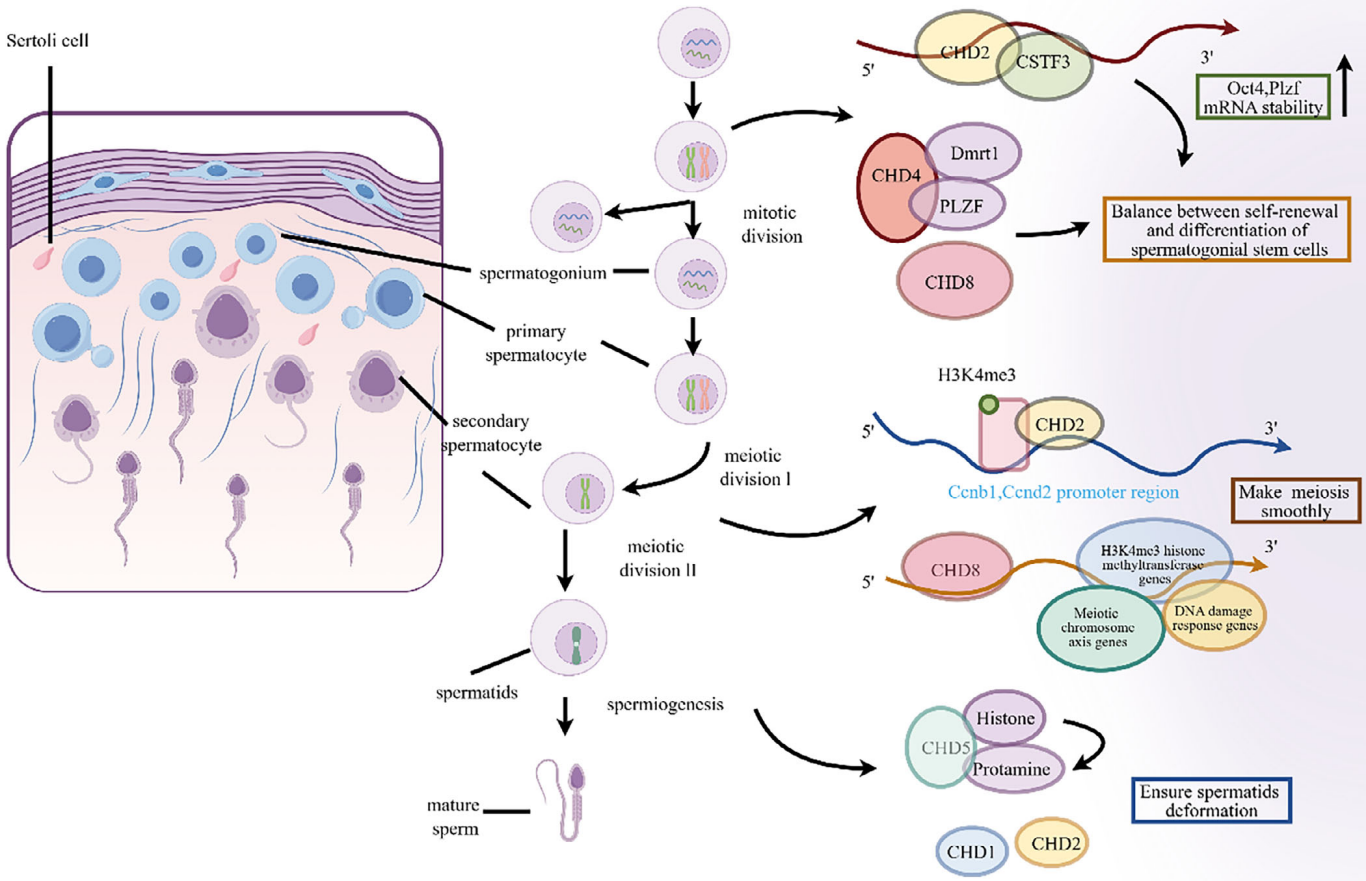
Roles and mechanisms of CHD proteins in spermatogenesis. Spermatogenesis can be primarily divided into three stages: spermatogonial proliferation and differentiation, spermatocyte meiosis, and spermatid metamorphosis and maturation. CHD2, CHD4, and CHD8 play roles in maintaining the self‐renewal and survival of SSCs. CHD2 and CHD8 ensure smooth progression of spermatocyte meiosis. CHD1 and CHD5 facilitate proper spermatid metamorphosis and ensure spermatid maturation. Some components of the model schematic were from www.figdraw.com, which was used for model drawing. Ccnb1, cyclin B1; Ccnd2, cyclin D2; CHD, chromodomain helicase DNA‐binding; Dmrt1, doublesex and mab‐3‐related transcription factor 1; PLZF, promyelocytic leukemia zinc finger protein.

Subfamily I, including CHD1 and CHD2, share DNA‐binding domains that demonstrate similar functions to SWI3 and adenosine deaminase 2 (ADA2) [15, 27]. CHD1 promotes spermatid metamorphosis, whereas CHD2 promotes spermatogonial survival and spermatocyte meiosis. CHD1 deficiency leads to hyperacetylation of histone H4, abnormal histone removal, and inadequate chromatin condensation, ultimately leading to sperm head malformations and residual cytoplasm, all of which contribute to the failure of spermatogenesis [28, 29, 30, 31]. CHD2 is highly expressed in male germ cells, particularly in meiotic phase I spermatocytes, where it maintains the enrichment of H3K4me3 at the promoter regions of cell cycle‐related genes such as cyclin B1 (Ccnb1) and cyclin D2 (Ccnd2), thereby promoting their transcription and the maintenance of an active chromatin state [25, 32, 33]. Additionally, CHD2 upregulates the expression of octamer‐binding transcription factor 4 (OCT4) and promyelocytic leukemia zinc finger protein (PLZF), two key transcription factors involved in spermatogenesis, by increasing mRNA stability through interaction with cleavage stimulation factor subunit 3 (CSTF3), maintaining the survival of spermatogonia [25, 34, 35] (Figure 1).

### 2.2 Role of the CHD3 Subfamily (CHD3–5) in Spermatogenesis

Subfamily II, including CHD3–5, contains the plant homeodomain (PHD) zinc finger domain, which promotes their binding to methylated histone residues and protein cofactors [15, 27]. CHD4 balances SSC self‐renewal and differentiation, whereas CHD5 promotes spermatid maturation. CHD4 maintains SSC survival by regulating the expression of doublesex and mab‐3‐related transcription factor 1 (Dmrt1) and PLZF [36]. During spermatogenesis, CHD4 is highly expressed in SSCs. Spermatogonium‐specific knockout of *CHD4* in neonatal mice results in the failure of survival of undifferentiated SSCs, thereby hindering early gamete development [37, 38]. Moreover, in male mice, germ cell‐specific knockout of *CHD4* leads to complete infertility, as indicated by the rapid loss of SSCs and excessive apoptosis [12, 39] (as shown in Figure 1).

In contrast, the specific function of CHD3 in spermatogenesis remains poorly defined and appears to be non‐essential in early stages. Although it is coexpressed with CHD4 in both germ and Sertoli cells, germ cell‐specific knockout of *CHD3* in mice does not disrupt spermatogenesis or fertility, suggesting functional redundancy or compensation by *CHD4* in maintaining the SSC pool [26, 37, 40]. However, both CHD3 and CHD4 are enriched in meiotic spermatocytes, particularly at the sex chromosome, suggesting potential specialized roles in meiosis [11]. CHD5 is detected exclusively in germ cells, suggesting a germ cell‐specific function for CHD5 [11]. The deletion of CHD5 can result in the immature exfoliation of sperm cells, disrupt the normal rhythm of the spermatogenic cycle, and induce abnormalities in sperm head morphology, ultimately leading to the failure of spermatogenesis [23, 41].

### 2.3 Role of the CHD7 Subfamily (CHD6–9) in Spermatogenesis

In subfamily III, which includes CHD6–9, the Swi3, Ada2, N‐CoR, TFIIIB (SANT) domain can promote non‐specific DNA binding [15, 42]. Among them, CHD8 has emerged as a critical regulator. Germ cell‐specific knockdown of *CHD8* in mice leads to progressive depletion of undifferentiated spermatogonia, impairs the formation of meiotic double‐strand breaks, and causes meiotic arrest and cell death, highlighting its essential role in maintaining the spermatogonial pool and supporting meiosis [43, 44, 45]. Mechanistically, CHD8 directly binds to and regulates the expression of key meiosis‐related genes, including those encoding H3K4me3 methyltransferases, meiotic adhesion proteins, and synaptonemal complex components [46, 47]. These findings collectively demonstrate that CHD8 sustains spermatogonial proliferation and survival while facilitating spermatocyte meiosis (Figure 1). In contrast, direct experimental evidence defining the roles of CHD6 and CHD9 in mammalian spermatogenesis is lacking.

### 2.4 Evolutionary Selection of Multiple CHD Remodelers in Spermatogenesis

Evolutionary selection for multiple, specialized CHD remodelers is driven by the extreme and stage‐specific chromatin remodeling demands of spermatogenesis. This is reflected in their non‐overlapping, stage‐specific expression: CHD4 functions predominantly in early SSC maintenance and meiosis, whereas CHD5 is expressed specifically in post‐meiotic round spermatids [11, 26]. This stage‐specific segregation indicates that a single remodeler cannot fulfill these distinct developmental requirements.

Critically, the ATPase domains of CHD4 and CHD5 share 60%–70% sequence similarity [48, 49], but they are not functionally interchangeable [50]. A compelling parallel comes from neurodevelopment, where CHD4 and CHD5 have been shown to act as mutually exclusive and non‐interchangeable catalytic subunits within the nucleosome remodeling and deacetylase (NuRD) complex; the loss of one cannot be functionally compensated by the other [51]. By analogy, it is plausible that in spermatogenesis, the distinct roles of CHD4 in SSC maintenance and CHD5 in spermatid maturation are equally irreplaceable, and that CHD5 overexpression would not rescue defects arising from CHD4 loss. This specificity is determined by their unique accessory domains and dedicated protein interactions: CHD4 operates primarily as the core ATPase of the ubiquitous NuRD complex [11, 52, 53], whereas CHD5 forms germ cell‐specific complexes with factors such as protamine chaperones [11, 41, 50].

From an evolutionary perspective, the testis is among the fastest evolving organs and is influenced by factors such as sperm competition [54]. Genes with reduced pleiotropic constraints in somatic tissues, such as *CHD5*, can thus undergo rapid functional innovation within the germline without compromising organismal viability [55]. This modular division of labor ensures reproductive resilience by preventing a deleterious mutation in one stage‐specific remodeler from catastrophically disrupting the entire spermatogenic program.

## 3 Molecular Mechanisms by Which CHD Proteins Regulate Spermatogenesis

CHD proteins orchestrate spermatogenesis through multiple interconnected molecular mechanisms, including chromatin remodeling, transcriptional regulation, epigenetic modification, and DNA damage repair. Dysregulation of any of these mechanisms can result in spermatogenic failure, as detailed in Table 2.

**TABLE 2:** Chromodomain helicase DNA‐binding (CHD) proteins regulate spermatogenesis via multiple molecular mechanisms.

Molecular mechanisms	Specific functions of CHD proteins in the molecular mechanisms regulating spermatogenesis	References
Chromatin remodeling	CHD1 promotes the deposition of histone variants, CHD4 maintains histone methylation or acetylation, and CHD5 regulates the transition from histone to protamine	[19, 23, 28, 37, 62, 63]
Transcriptional regulation	CHD2 maintains H3K4me3 enrichment in the promoter regions of cell cycle genes, CHD4 interacts with the NuRD complex and the GDNF‐response transcription factor SALL4, and CHD5 regulates the expression of transcription protein and protamine	[23, 24, 25, 43, 63, 65, 67, 68, 69, 104, 105, 106]
Epigenetic modifications	CHD4 recruits histone‐modifying enzymes; CHD5 regulates histone acetylation and DNA methylation	[23, 37, 41, 72, 73]
DNA damage repair	CHD4 mediates DSB repair via homologous recombination, CHD7 facilitates the assembly of the NHEJ repair pathway	[77, 79, 82, 85]

Abbreviations: GDNF, glial cell line‐derived neurotrophic factor; NuRD, nucleosome remodeling and deacetylase; SALL4, Sal‐like 4.

### 3.1 Chromatin Remodeling

CHD proteins, as ATP‐dependent chromatin remodeling factors, play essential roles in spermatogenesis by orchestrating the histone‐to‐protamine transition, promoting histone variant deposition, and maintaining specific histone modifications required for normal progression [56, 57, 58, 59]. CHD1 regulates chromatin structure by promoting H3.3 variant deposition to control gene expression [28]. As a core component of the NuRD complex, CHD4 ensures proper chromatin remodeling through coordinated acetylation and methylation, which is critical for SSC survival and testicular development [37, 60, 61, 62]. CHD5 directly participates in the histone‐to‐protamine replacement process and maintains DNA integrity during spermiogenesis [23, 63, 64].

### 3.2 Transcriptional Regulation

CHD proteins regulate transcription throughout spermatogenesis and control key genes involved in SSC self‐renewal and differentiation, spermatid maturation, and sperm chromatin remodeling. CHD2 regulates SSC self‐renewal by maintaining H3K4me3 enrichment in the promoter regions of cell cycle genes, thereby promoting their transcription [25, 65, 66]. CHD4 interacts with the NuRD complex and the glial cell line‐derived neurotrophic factor (GDNF)‐response transcription factor Sal‐like 4 (SALL4) to regulate the expression of self‐renewal genes and precursor cell signature genes, thereby maintaining the SSC pool and influencing cell fate decisions [67, 68, 69]. CHD5 plays a role in regulating the expression of transcription proteins (Tnp1/Tnp2) and protamines (Prm1/Prm2) [23, 63, 70]. CHD8 deficiency leads to impaired spermatogonial proliferation and abnormal meiosis, likely because of its regulation of key gene transcription [43, 71].

### 3.3 Epigenetic Modification

CHD proteins regulate various epigenetic modifications, including histone methylation and acetylation, histone variant deposition, and DNA methylation. CHD4 interacts with histones through its chromodomain to recruit histone‐modifying enzymes, thereby regulating gene transcription [37]. CHD5 significantly influences histone H4 acetylation levels during spermatogenesis; its deletion leads to a decrease in H4 acetylation, suggesting that CHD5 may participate in sperm chromatin remodeling by regulating histone acetylation [23, 41, 72]. Notably, smoking may affect sperm quality by altering the methylation pattern of the *CHD5* gene, and increased CHD5 methylation may be involved in the regulation of DNA methylation, which in turn affects spermatogenesis [73, 74, 75].

### 3.4 DNA Damage Repair

CHD proteins maintain genomic stability during spermatogenesis by promoting the recruitment of repair factors, regulating chromatin structure, and participating in specific repair pathways [22, 76]. CHD3.1 maintains heterochromatin stability but temporarily suspends its compacting function during DNA damage to promote the feasibility of repair [77, 78]. CHD4 is a core chromatin remodeling factor involved in the DNA double‐strand break repair response. It is rapidly recruited to DSB sites, where it facilitates local chromatin relaxation and contributes to homologous recombination‐mediated DSB repair [77, 79]. CHD5 maintains genomic integrity through p53 pathway modulation rather than direct DSB repair, determining cell fate (cell cycle arrest or apoptosis) following DNA damage [80, 81, 82]. CHD6 contributes to genomic stability by protecting cells from oxidative damage, a process that may indirectly support the DSB repair process [82, 83, 84]. CHD7 interacts with poly (ADP‐ribose) polymerase 1 (PARP1) to promote chromatin expansion at DSB sites, thereby facilitating the assembly of the non‐homologous end joining repair pathway, indicating its role in maintaining sperm DNA integrity by regulating chromatin structure [85].

### 3.5 Integrated Coordination of CHD Protein Networks

The discrete molecular functions described above are not executed in isolation but are dynamically interwoven into a functional network that ensures robust spermatogenesis. This integration manifests at multiple levels: (1) mechanistic coupling: Individual CHD proteins, such as CHD4 within the NuRD complex, directly couple ATP‐dependent chromatin remodeling with histone deacetylation to repress differentiation genes [24, 53, 86, 87]. Similarly, CHD5 coordinates a sequential program in which its remodeling activity at histone–protamine transition sites is functionally coupled with the transcriptional regulation of protamine genes (Prm1/Prm2), ensuring proper chromatin compaction [23, 41]. These examples illustrate that for CHD proteins, structural chromatin remodeling and transcriptional control are two inseparable facets of a unified regulatory event. (2) Signal integration: CHD proteins serve as nodes that integrate intrinsic genetic programs with extrinsic environmental cues, as exemplified by smoking‐induced CHD5 hypermethylation, which links environmental exposure to epigenetic and functional dysregulation [73]. (3) Spatiotemporal orchestration: The stage‐specific expression and specialized functions of CHD family members—from CHD4 maintaining genome stability in mitotic and meiotic cells [24] to CHD5 executing terminal chromatin compaction [41]—form a choreographed handoff, ensuring process continuity [26]. Consequently, CHD protein dysfunction does not merely disrupt a single pathway; it perturbs this integrated network, leading to the systemic failure characteristic of spermatogenic arrest. This network perspective underscores why therapeutic strategies may need to consider network resilience rather than individual components.

## 4 Relationships Between CHD Proteins and Male Infertility

### 4.1 *CHD* Gene Mutations and Male Infertility

There is a strong association between genetic mutations in *CHD* genes and male infertility. Genome‐wide association studies (GWASs) have revealed that polymorphisms in the *CHD2* gene are linked to an increased risk of non‐obstructive azoospermia (NOA) [88, 89, 90]. Moreover, polymorphisms in the *CHD5* gene may be genetic risk factors for both NOA and severe oligospermia. Specifically, a polymorphism in the G allele of the *CHD5* gene, rs9434741, increases the risk of infertility by 1.52‐fold, with the GG genotype being significantly associated with infertility risk [91, 92, 93]. Missense mutations in the *CHD7* gene have been associated with clinical features observed in infertile men, such as male hypogonadotropic hypogonadism (IHH) [94, 95, 96]. These findings indicate that *CHD* gene mutations can contribute to male infertility by affecting spermatogenesis.

### 4.2 CHD Proteins as Potential Targets for the Diagnosis and Treatment of Male Infertility

#### 4.2.1 Diagnostic Potential: CHD5 Methylation as a Non‐Invasive Biomarker

Bioinformatics analysis revealed that CHD5 may be a potential biomarker for NOA [97]. Notably, the methylation level of the *CHD5* gene can serve as a biomarker to assess semen quality and identify risk factors for infertility [73]. Recent studies have demonstrated that CHD5 methylation status in blood samples is significantly correlated with semen parameters (*p* < 0.001), with hypermethylation associated with reduced sperm concentration [73]. This epigenetic signature offers a non‐invasive diagnostic approach for evaluating male fertility status, as methylation analysis can be performed on peripheral blood without the need for testicular biopsy. Furthermore, CHD5 methylation levels may serve as a predictive biomarker for environmental risk factors, for example, smoking as smokers exhibit higher CHD5 methylation levels (22.5%) than non‐smokers do (14.6%), accompanied by decreased sperm concentrations (32.21  ± 5.27 vs. 55.27  ± 3.38 × 10^6^/mL) [73].

#### 4.2.2 Therapeutic Challenges and Strategies for Specificity

Targeting CHD proteins therapeutically faces two major hurdles: First, their ubiquitous expression and potential toxicity merit attention because of their essential functions in diverse tissues, which increase the risk of off‐target effects from systemic inhibition [50, 98]. Second, the blood–testis barrier limits drug delivery to germ cells [99].

Strategies to achieve cell‐type specificity include the following: (1) exploiting stage‐specific expression: The distinct expression peaks of CHD4 (in SSCs) and CHD5 (in round spermatids) [11, 26] provide a window for stage‐selective intervention; (2) development of local delivery systems: Direct intratesticular injection (e.g., via lipid nanoparticles) can increase testicular bioavailability while minimizing systemic exposure [100, 101].

The translation of targeted therapies for CHD proteins may require a combination strategy to overcome specificity barriers. For instance, the design of nanocarriers capable of stage‐specific, triggered drug release in response to molecular signatures of germ cells may be a promising direction. However, the efficacy and long‐term safety of such integrated approaches must undergo comprehensive pre‐clinical evaluation before clinical translation can be considered.

## 5 Conclusions and Future Perspectives

CHD proteins play significant roles in regulating spermatogenesis. CHD2 maintains SSC self‐renewal by increasing chromatin accessibility and mRNA stability. CHD4 is essential for maintaining the SSC pool, as it regulates the expression of self‐renewal genes and precursor genes. CHD5 facilitates the histone‐to‐protamine transition and chromatin remodeling during spermatogenesis. Mutations in *CHD7* are associated with clinical features of male infertility. CHD8 contributes to the maintenance of normal proliferation and apoptosis in spermatogonia and promotes meiotic progression. Overall, CHD family proteins perform important regulatory functions in multiple stages of spermatogenesis that are vital for maintaining normal spermatogenesis and male fertility.

Despite significant progress, there are several limitations that exist in our current understanding of the roles of CHD proteins in spermatogenesis. First, most studies have focused on a limited number of CHD family members, whereas the roles of other members have been largely unexplored. Second, existing studies predominantly rely on animal models, particularly mouse models, and further validation is needed to determine the relevance of the findings to human spermatogenesis. Additionally, the interactions between CHD proteins and other epigenetic regulators, as well as their synergistic effects on spermatogenesis, need to be more thoroughly investigated. Future research should focus on the following goals: (1) systematic investigation of the roles and regulatory mechanisms of all CHD family members in spermatogenesis to elucidate the commonalities among different members. (2) Validation of the roles of CHD proteins in human spermatogenesis via human samples and clinical data. (3) Exploration of the interaction network between CHD proteins and other epigenetic regulators. (4) Development of diagnostic and therapeutic strategies based on CHD proteins for the clinical management of male infertility. These studies improve our understanding of the roles of CHD proteins in spermatogenesis and provide new insights into the diagnosis and treatment of male infertility.

## Author Contributions

The work presented here was conducted in collaboration with all the authors. Mingrui Zhang wrote the main manuscript, and Guoqiang Liu and Minggang Zhu contributed to the discussion. Mingrui Zhang, Di Liu, and Feng Pan drafted the manuscript. Feng Pan provided editorial suggestions and final revision of the manuscript. All the authors were involved in reviewing and commenting on the manuscript and gave their approval of the submitted version of the manuscript.

## Funding

This work was supported by a grant from the National Nature Science Foundation of China (Grant 81873854) and the Open Project of Hubei Jiangxia Laboratory (Grant 2025JXKF018).

## Conflicts of Interest

The authors declare no conflicts of interest.

## Data Availability

Data sharing is not applicable to this article as no new data were created or analyzed in this study.
